# A Longitudinal Study of the Effects of Service-Learning on Physical Education Teacher Education Students

**DOI:** 10.3389/fpsyg.2022.787346

**Published:** 2022-03-16

**Authors:** María Maravé-Vivas, Jesús Gil-Gómez, Teresa Valverde-Esteve, Celina Salvador-Garcia, Oscar Chiva-Bartoll

**Affiliations:** ^1^Department of Pedagogy and Didactics of the Social Sciences, Language and Literature, Universitat Jaume I, Castellón, Spain; ^2^Department of Didactics of Music, Visual and Body Expression, University of Valencia, Valencia, Spain; ^3^Facultad de Educación, Universidad Internacional de la Rioja, Logroño, Spain

**Keywords:** service-learning, physical education teacher education, civic skills and attitudes, pedagogical model, longitudinal study

## Abstract

Research examining Service-Learning (SL) in Physical Education Teacher Education (PETE) is ample. However, long-term investigations are still scarce and literature demands the application of this type of design to uncover the effects of SL on the long run. This study followed a longitudinal quantitative approach; thus, the participants completed the Civic Attitudes and Skills Questionnaire (CASQ) in three occasions (pretest-postest1-postest2). Results show that there exist significant differences between mean values of the global outcomes of the CASQ; concretely, there was an improvement in the first interval followed by a decrease in the second period. Regarding the different dimensions of the CASQ, leadership skills, attitudes towards social justice and attitudes towards diversity showed significant differences too. This research leads towards better understanding of methodological strategies promoting quality education, positing SL as an adequate possibility in this respect, also in the long term.

## Introduction

The transition towards the use of active methodologies in higher education during the last decade (McAleese, 2013, 2014) has generated multiple experiences involving educational innovation as well as related research examining their effects. Within the wide spectrum that these methodologies encompass, the present work focuses on Service-Learning (SL). There are a number of definitions for this pedagogical method. However, the essential traits that all of them highlight share the idea of SL being a methodology through which students taking a subject participate in an activity involving a service. This service is aimed at satisfying a community need. In addition, SL comes with an essential reflective process in order to facilitate comprehensive understanding. In this sense, the role of university faculty is critical to ensure intentional and multidirectional reflective processes by inquiring about the issues that occur in the realities in which students work thus fostering learning (Bringle and Hatcher, 1995). This approach leads students to acquire not only academic contents, but also other types of learning derived from the interaction with the context and the direct contact with service recipients. In the teacher training-specific field, different reviews (Conway et al., 2009; Celio et al., 2011; Salam et al., 2019) identify that, besides of developing academic competencies, SL promotes personal, social and civic competences among physical education teacher education students (PETEs). In our opinion, from a pedagogical perspective, these ideas are critical in the teacher training arena, since PETEs must acquire an integral training that provides them with sufficient knowledge to give adequate educational responses in their professional future.

The aforementioned information is closely aligned with the Sustainable Development Goals (SDG), particularly with SDG number 4, which is focused on quality education promotion. According to the definition of such a goal, it strives to ensure inclusive and equitable quality education and promote lifelong learning opportunities for all (United Nations, General Assembly, 2014). We do consider teacher training to be critical to move forward steadily and decidedly to achieve this goal. Without doubt, properly attending the diversity in schools is one of the challenges related to SDG 4. In this sense, the teacher training period is decisive in order to provide PETEs with opportunities to work directly with diverse populations and experiences, promoting their civic attitudes and skills; because these will frame their backgrounds and adopt inclusive approaches in their future lessons.

At the higher education stage, there exist multiple possibilities to prepare SL programmes aligned with SDG (Castro et al., 2020), also in the teacher training arena (Byker and Ezelle-Thomas, 2021). For example, Daum et al. (2021) state that PETEs should interact and work hand in hand with children of different cultural backgrounds and diverse characteristics in order to enhance teachers being more culturally responsible. Preparing teachers to develop civic attitudes and skills is the first step for them to be transmitted to future generations. Therefore, SL may emerge as a powerful tool in the teacher training field, since it favours the flourishing of civic attitudes and skills that facilitate a peaceful coexistence in a globalised and multicultural system, which is one of the challenges faced by current European higher education (McIlrath et al., 2019).

SL is having a great reception and expansion in the field of Physical Education (PE; Chiva-Bartoll and Fernández-Río, 2021). In this area, previous studies have linked the effects coming with SL to SDG, arguing that this methodology can contribute significantly in the development of ‘socially critical professionals’ (García-Rico et al., 2021). Furthermore, other studies suggest that SL may increase civic compromise and responsibility of the participating students (Richards et al., 2015; Meyer et al., 2016; Caspersz and Olaru, 2017); their civic competence (Gil-Gómez et al., 2016); their intercultural competence (Johnson and Howell, 2017; Chiva-Bartoll et al., 2021a); as well as promote social justice (Moely et al., 2002; Asghar and Rowe, 2017).

Other positive effects for students engaging in SL encompass the development of civic and political identity, citizenship understanding, responsibility upon their own actions and a better awareness about themselves in comparison with other people and their communities (Iverson and James, 2013). In addition, PETEs may increase their effective personality (Chiva-Bartoll et al., 2019); develop more positive attitudes towards special education needs (Wilkinson et al., 2013; Chiva-Bartoll et al., 2021b); as well as civic skills and attitudes (Maravé-Vivas et al., 2019), being this last dimension primary for the present study. Nevertheless, the majority of the investigations examining SL in the PE field use short-term designs. In this sense both, systematic reviews and specific literature highlight the need to carry out studies considering the long-term effects of this methodology to examine whether the effects that SL entails are long-lasting on the participant students (Maloney and Griffith, 2013; Chiva-Bartoll et al., 2019; Ruiz-Montero et al., 2019; Seban, 2019; Pérez-Ordás et al., 2021).

As mentioned before, one of the effects coming with the application of SL is linked to the development of civic skills and attitudes on the part of the participant PETEs (Maravé-Vivas et al., 2019). Thus, actually, civic learning is considered to be an essential element of SL (Bringle and Clayton, 2021). At the same time, according to Arroyo-Resino et al. (2021), civic knowledge is linked to quality education, the fourth SDG. In addition, the International Civic and Citizenship Education Study^1^ investigates the ways in which young people are prepared to undertake their roles as citizens in the world and reports on students’ knowledge and understanding of concepts and issues related to civics and citizenship. And, in fact, this study has been recognised by UNESCO as a solid evidence in monitoring progress towards SDG 4. Consequently, it is not surprising that authors such as Maurissen et al. (2018) accentuate the need to promote school contexts characterised by fairness and responsiveness.

In order to establish this type of school contexts, the teachers’ task is of utmost importance, and previous teacher training should ensure adequate opportunities to promote civic skills and attitude on PETEs, as SL does (Maravé-Vivas et al., 2019). However, these skills and attitudes should remain over time so that SL participants can apply them in the future, when they will be working in schools. For this reason, it is necessary to examine if this learning persists after having participated in SL (Maloney and Griffith, 2013; Chiva-Bartoll et al., 2019; Ruiz-Montero et al., 2019; Seban, 2019; Pérez-Ordás et al., 2021). In order to fill this gap, the present text aims to examine the effects of SL on the development of civic skills and attitudes of university students, as well as their perdurability 2 years after its completion.

## Materials and Methods

### Study Design

**Figure 1:**
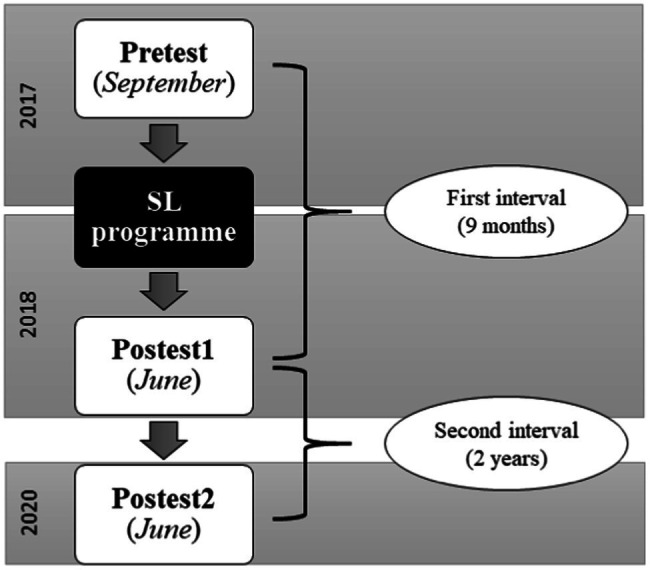
Longitudinal design with three measures over time.

The present study followed a longitudinal quantitative approach, and three measures over time (pretest-postest1-postest2) were taken to analyse the evolution of the students’ civic attitudes and skills. An overview of the research is shown in Figure 1.

**Figure 2:**
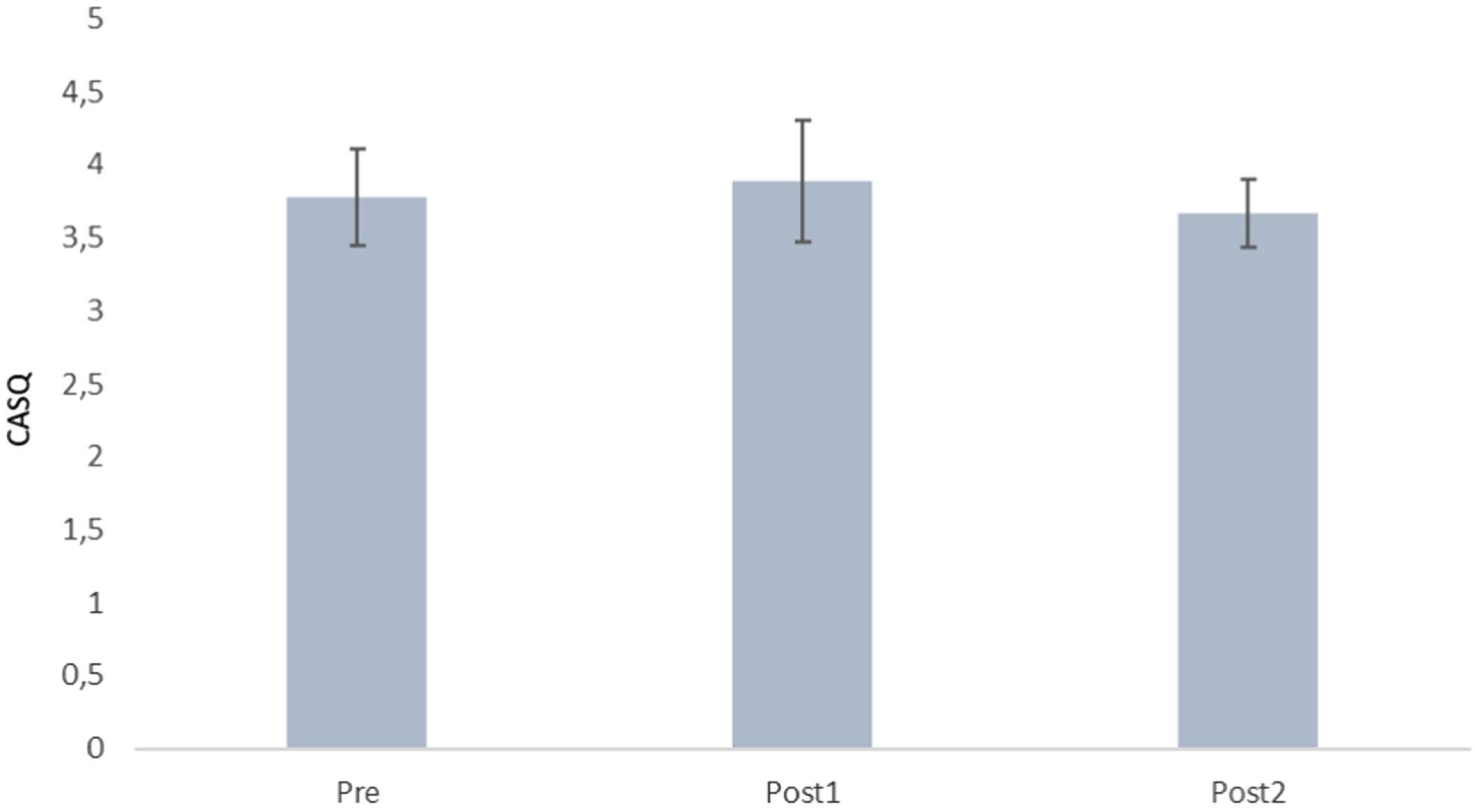
Descriptive values of the CASQ.

### Hypotheses

Considering the objective of this study, two hypotheses were established as:

*Hypothesis 1*: Students participating in the SL programme will obtain significant improvements in the civic skills and attitudes.

*Hypothesis 2*: The acquired levels of civic skills and attitudes will remain two years after their participation in the programme.

### Participants

The recruitment of participants followed a non-probabilistic sampling since a natural group formed by students who belonged to the second year of the Bachelor’s Degree in Early Childhood Education took part in the intervention programme. The sample of the pretest consisted of 123 students, in 2017. The number of participants was reduced in the postest1 to 98 students, in 2018. Finally, in the postest2, 54 students participated in 2020. This is a typical limitation of longitudinal studies, since they entail higher drop-out rates (Deeg et al., 2002; Delgado and Llorca, 2004). These participants were reduced because of a lack of response to the emails and phone calls by the research team.

### Instruments

The instrument used in this research was the Civic Attitudes and Skills Questionnaire (CASQ), validated by Moely et al. (2002). It has 44 items that measure civic attitudes and skills, and analyses six dimensions: (1) civic action (future intention to get involved in some service to the community), (2) development of interpersonal and problem-solving skills (ability to listen, work cooperatively, make friends, put oneself in someone else’s shoes and develop logical and analytical thinking to solve problems), (3) political awareness (knowledge of local and national political issues and events), (4) leadership skills (ability to be effective as leaders), (5) attitudes towards social justice (attitudes about poverty and the misfortune of others, as well as about the ability to propose solutions) and (6) attitudes towards diversity (appreciation and appreciation of relationships with people of different origins and characteristics). Some examples of items are as: ‘I can successfully resolve conflicts with others’, ‘I am aware of the events that happen in my local community’, ‘I have the ability to lead a group of people’ or ‘We need to change people’s attitude to solve social problems’. Its internal consistency is *α* = 0.88 (Moely et al., 2002), while in our case, the results were *α* = 0.634 and it is answered through a Likert scale with scores from one to five, where one represents the position ‘not at all agree’ and five represents ‘completely agree’. The result for each construct corresponds to the mean value of the items that are included.

### Intervention Programme

The participants of the study were taking a subject in the field of PE within the Bachelor’s Degree in Early Childhood Education offered at Universitat Jaume I, in which the SL was applied. Table 1 shows a description of the SL programme.

**Table 1:** Description of service-learning programme.

Service-Learning programme	
Subject: Fundamentals of Corporal Expression; Motor Games in Early Childhood Education.	Compulsory subject within the Degree of the Didactics of Corporal Expression area. It is an annual course, with a load of six ECTS credits (150 h).
Learning objectives	-Learning how to use the knowledge related to motor and expressive activities in the form of play adapted to user reality in specific situations. -Applying civic skills and attitudes aimed at improving personal interaction. -Modifying the PETEs perception of diversity through coexistence and reflection. -Improving children’s motor skills and socialisation.
Service objectives	-Planning and executing motor games and body expression sessions with the aim of helping to improve motor development, body expression and inclusion of children with functional diversity.
Formation of groups	Groups of four to five people
Participating entities All of them are characterised by caring for children with functional diversity and/or problems of social exclusion.	AAC APADAHCAS APNAC CEIPC CEEPR CEIPTB
Phases applied by students	Initial motivation; diagnosis; design and planning; implementation and celebration; closure. Model proposed by CLAYSS (2016)
Amount of service hours of service	25 h on average

AAC, Asociación Asperger de Castelló de la Plana (Asperger Association of Castelló de la Plana); APADAHCAS: Asociación de Padres de Afectados por Déficit de Atención e Hiperactividad de Castellón (Parents’ Association of Children with Attention Deficit and Hyperactivity of Castellón); APNAC, Asociación de Padres de Niños Autistas de Castellón (Parents’ Association of Children with Autism of Castellón); CEIPC, Colegio de Educación Infantil y Primaria Cervantes de Castelló de la Plana (Cervantes kindergarden and primary school of Castelló de la Plana); CEEPR, Centro de Educación Especial Penyeta Roja (Penyeta Roja Special Education School); and CEIPTB, Colegio de Educación Infantil y Primaria Tombatossals de Castelló de la Plana (Tombatossals kindergarden and primary school of Castelló de la Plana).

### Data Analysis

The data analysis was carried out in a descriptive (mean ± standard deviation) and inferential way. After verifying the normal distribution of the results of que CASQ (Kolmogorov Smirnov), we carried out a repeated measures ANOVA analysis (CASQ questionnaire) in three different time frames (Pretest × Postest1 × Postest2), consistent with the verification of the hypotheses and according to the examined longitudinal study. Subsequently, a comparison of the Pretest, Postest1 and Postest2 values was carried out through Bonferroni’s post-hoc, to determine between which samples significant differences appear. In both analyses, we considered those differences significant when *p* ≤ 0.05. We added the analysis of the effect size calculating the Eta squared and Cohen’s d, considering the latter value according to the Sawilowsky scale (2009): very low (*d* = 0.01), low (*d* = 0.2), medium (*d* = 0.5), strong (*d* = 0.8), very strong (*d* = 1.2), and huge (*d* = 2.0). We also obtained the values of the Confidence Interval (ICC95%). This analysis was carried out using SPSS software (V26, Chicago, Il, United States).

### Ethical Considerations

All the participants involved received thorough information about ethical considerations regarding informed approval and confidentiality, building on guiding principles from the University ethics committee and had thereafter accepted to take part in this study.

## Results

First, the descriptive values obtained from the CASQ questionnaire globally are presented in Figure 2 differentiating three different measures: prior to the intervention (Pretest), 9 months later, when the intervention finished (Postest1); and 24 months after the end of the intervention (Postest2).

Next, Table 2 shows the variance of each of the six constructs of the CASQ.

**Table 2:** Descriptive analyses by dimensions of the CASQ.

*Dimensions*	*Pretest (mean ± SD)*	*Postest1 (mean ± SD)*	*Postest2 (mean ± SD)*	*Sig*
Civic action	3.67 ± 0.53	3.77 ± 0.70	3.73 ± 0.69	0.103
Interpersonal skills	4.32 ± 0.40	4.43 ± 0.42	4.43 ± 0.35	0.321
Political awareness	3.04 ± 0.56	3.08 ± 0.60	3.18 ± 0.54	0.190
Leadership skills	3.03 ± 0.64	3.19 ± 0.70	3.32 ± 0.39	0.032
Attitudes towards social justice	4.12 ± 0.42	4.27 ± 0.47	3.42 ± 0.32	0.000
Attitudes towards diversity	3.78 ± 0.61	3.86 ± 0.62	3.11 ± 0.37	0.000

Considering the six dimensions of the CASQ, Bonferroni’s post-hoc analysis showed that there were no significant changes among the results in the civic action, interpersonal skills and political awareness results These results differ for the other dimensions (leadership skills, attitudes towards social justice and attitudes towards diversity), which showed significant differences. Bearing in mind these results, it is interesting to determine in which specific measurements do such differences appear.

Focusing on the values presenting significant differences, the values of the leadership construct were slightly higher in Postest1 and Postest2. Significant differences were obtained between the Pretest and Postest2 (*F*_2, 159_ = 3.330; *p* = 0.038; *d* = −0.54; IC: −0.567, −0.017; *η*^2^: 0.040).

The mean value of the dimensions of attitudes towards social justice increased in Postest1 but decreased in Postest2. In any case, results show statistically significant differences when comparing Pretest with Postest1 (*p* < 0.000; *d* = 0.336; IC: −0.89, −0.508), Postest1 with Postest2 (*p* < 0.00; *d* = −2.114; IC: −1.04, −0.656) and Pretest with Postest2 (*F*_2, 159_ = 65.512; *p* < 0.001; *η*^2^: 0.452).

The attitude towards diversity dimension also increased in Postest1, but decreased in Postest2, showing statistically significant differences (*F*_2, 159_ = 31.438; *p* < 0.000; *η*^2^: 0.283). The same effect occurs when comparing Pretest with Postest1 (*p* < 0.000; *d* = −1.469; IC: −1.004, −0.499) and Pretest with Postest2 (*p* = 0.000; *d* = 0.130; IC: −0.926, −0.421). Both in this dimension and in the previous one, there are significant differences in each of the study measurements.

## Discussion

This study aimed to analyse the effects of a SL programme applied in the PE field on civic attitudes and skills of the participating PETEs. The formulated hypotheses considered that participating in the SL programme would produce a significant improvement in the civic skills and attitudes of the students (H1), and these effects on civic skills and attitudes would remain 2 years after participation in the programme (H2).

The results obtained through the ANOVA analysis show that, from a global perspective, there were significant differences in the mean values of the CASQ. The enhancement of students’ civic attitudes and skills in the first interval (between the start and the end of the SL programme) may be explained as a consequence of the students’ experiences when interacting and working hand in hand with children with special education needs (An and Decker, 2019). In this sense, according to Mansfield et al. (2012), when students have a voice in designing curriculum, which is an intrinsic characteristic of SL, they develop democratic civic habits that become embedded in their psyche. Therefore, since SL is usually conducted in collaboration with one or more community organisations, as it was the case of the present study, Adler and Goggin (2005) claim that this methodology comes with the opportunity to foster ‘civic learning’ about social responsibility. Similarly, Caspersz and Olaru (2017) carried out an investigation to understand the value of SL examining students’ viewpoints, and they found out that besides to offering a potentially transformative learning experience, SL emerged as a process of learning how to create social change and develop the personality and social skills of the learners.

The evolution of pre-service teachers’ personality is important and, in the case of the present study, seems a consequence of applying helping behaviours, understanding and trying to organise actions to enhance the children and their families’ personal situation. In brief, understanding better what social justice is as a consequence of taking part in real experiences with social disadvantaged groups that comes with SL (García-Rico et al., 2020). These results are aligned with a number of studies that also found that students’ outcomes related to these areas improved after participating in SL programmes within the field of PE (Whitley et al., 2017; An, 2019; Maravé-Vivas et al., 2019).

In any case, what is noteworthy here is the decrease of civic attitudes and skills in the second interval (between postest1 and postest2). Specialised literature suggests that these results are inconsistent, since they suggest that these attitudes and skills remain not only 2 years, as in this study, but even for longer periods of time (Astin et al., 1999; Vogelgesang and Astin, 2000; Astin et al., 2006; Vogelgesang, 2009; Fullerton et al., 2015).

Two reasons might justify the findings of the present research. On the one hand, SL is context-led and it is inextricably linked to culture and traditions of the community in which it is applied. However, there are no previous longitudinal studies in our context, therefore comparing our results with those of other settings might not be the best option. This fact encourages implementing further investigations in this line to deepen in the understanding of SL effects on complex aspects such as personality, behaviours, attitudes and teacher identity (González, 2013).

On the other hand, and probably more powerful, university students might have stronger expectancies when their critical awareness flourishes. To our understanding, after participating in SL, they have much more information about diversity, exclusion and the problematics suffered by socially disadvantaged people. When time goes by and students can contrast the reality to their previous experiences, they improve their awareness regarding the long way to reach real inclusion (Ngwenya et al., 2021). This may be demotivating, pull apart their expectance and, as a consequence of finishing the SL programme (the generator stimulus), they may have more focused responses on their limitations regarding civic attitudes and skills, as well as their self-efficacy to properly attend diversity. In this sense, studies that have applied an initial training focusing on inclusion completed with a second intervention later on, found out that there were gains in teachers’ self-efficacy due to the first programme carried out, and that these gains remained afterwards thanks to a second enrichment programme applied (Mintz, 2019). In any case, an interesting field of research may focus on these ideas.

In order to delve into the results of this study, the outcomes regarding each of the dimensions of the CASQ are also discussed. The ANOVA carried out shows that three dimensions did not show statistically significant differences between measures (civic action, interpersonal skills and political awareness). The evolution of the mean scores (Table 2) displayed subtle improvements in the other three dimensions. This means that there is a slight increase in the first interval (pretest-postest1), and this effect continues in the second interval (postest1-postest2), although no significant differences were reached.

Both, civic action and political awareness are closely linked to civic attitudes, fostering social empowerment and participation. They may range from understanding the need with which students are working to understanding how entities attend such needs. In this sense, according to Chiva-Bartoll et al. (2021a), curricula must be connected to society through an explicit compromise towards inclusivity and social transformation. For the majority of the students taking part in the SL programme, this is the first time they face such a real situation entailing social participation, and this requires a process of internalisation. Probably, this fact is the reason explaining the subtle increases obtained in the study, which is a possibility supported by previous investigations that display improvements too (Astin et al., 1999, 2006; Vogelgesang, 2009; Iverson and James, 2013 Fullerton et al., 2015; Moely and Ilustre, 2016).

The evolution of interpersonal skills slightly increased, reporting no significant differences. Specifically, there was an improvement in the first interval. This improvement remained stable in the second interval despite presenting a high mean value (4.32 points). Some authors (Keen and Hall, 2009 Moely and Ilustre, 2016; Whitley et al., 2017) reported an enhancement in interpersonal skills, which are consistent with our results. Working with children with functional diversity requires the development of communicative, listening and interaction skills; since university students must work together to plan the sessions and be in contact with the children’s relatives. At this point, it is relevant to remember that providing a service consists of planning and carrying out motor games and body language sessions. This is not an easy task, because university students have to adapt the activities and tasks to the specific features of the children they are working with. This challenge comes with the benefit of promoting their problem-solving capabilities, which are a nuclear part of interpersonal skills. Nowadays, teachers are expected not only to manage the conflicts that may emerge in the classroom, but also to teach students how to manage them by themselves (Pina et al., 2021). Therefore, any related learning in this respect owes a tremendous value. The participant pre-service teachers have evolved in this respect, as it happened before in the study performed by Miller and González (2019).

Bonferroni’s post-hoc statistic reported statistically significant differences in the dimensions focused on social justice and attitudes towards diversity on both the first interval (pretest-postest1) and second interval (postest1-postest2). Nevertheless, these differences did not display improvements in all the cases. In addition, regarding the leadership dimension, although mean scores increased steadily, significant levels were only achieved between pretest and postest2.

YorkBarr and Duke (2004) claim that teachers’ leadership is the process through which they are able to influence other teaching staff or member of the educational community with the aim of improving teaching practices. Childs-Bowen et al. (2000) consider teachers to be leaders when students’ learning is affected, since there is a contribution towards school’s enhancement and other agents are empowered to participate in actions fostering educational improvement. Consequently, it is fundamental to apply methodologies which promote leadership skills in teacher training programmes. In the present study, applying SL entailed steady and significant long-term improvements, in accordance with the results obtained by Moely and Ilustre (2016). This improvement is not surprising since university students had to face a real teaching experience dealing with children with functional diversity. Indeed, leadership skills were necessary for the sessions to be successful.

Concerning attitudes towards social justice dimension, scores display a significant improvement in the first interval (pretest-postest1). This means that university students increased their attitudes when they were participating in the SL programme. According to Jordan et al. (2009), teachers should be able to promote social inclusion among their students. Delving into these ideas, Martínez-Usarralde and Chiva-Bartoll (2020) claim that one of the key roles that SL may play lies in its capacity to generate awareness among the participants and prepare them to identify and face social injustice in the future. The results obtained in the present investigation respond to these ideas and show that SL may be a short-term adequate tool, in accordance with the studies of Chambers (2017) and Whitley et al. (2017). The doubt emerges when focusing on the long-term outcomes. Although authors such as Moely and Ilustre (2016) state that the effects were perdurable, the present investigation found a significant decrease in the attitudes towards social justice dimension in the second interval (postest1-postest2). In other words, on this occasion, the effects did not remain 2 years after the SL intervention. A possible explanation may be the absence of stimulus (interaction with the children with functional diversity) and the end of the university experience itself. In order to counteract this effect, SL could be optimised, reflection phases could be intensified, and this methodology could be extended to other subjects within the same degree. These ideas open a suggestive line of research that should be tackled in the near future.

This same effect also appears in the attitudes towards diversity dimension. Without doubt, and according to current legislative frameworks, this dimension is critical in teacher training. Teachers should be able to teach how to coexist in multicultural classrooms, which is a reality in current schools. In our SL programme, university students were in direct contact with this type of scenario, where a variety of problems related to functional diversity tend to appear. The novelty, the emotions experienced, or the feedback exchanged with their peers and professors may explain the improvements in the first interval, which are similar to those obtained by Wilkinson et al. (2013). Regarding the longitudinal outcomes, again, our results differed from those of Moely and Ilustre (2016), because the attitudes towards diversity did not remain in the second interval (postest1-postest2). Probably, more exposure time with the stimulus and more coordination among university professors in terms of methodology are needed. In addition, we encourage scholars to keep on performing longitudinal studies focusing on the effects of SL in order to reach a better understanding of its impact.

According to the present discussion and the conclusions obtained from it, the first hypothesis was accepted, since the improvement in the civic skills and attitudes in the students could be due to their participation in the SL programme. In addition, hypothesis 2 is partially accepted because some dimensions related to civic skills and attitudes were maintained 2 years after the SL programme.

Bearing in mind the scarcity of longitudinal studies examining the effect of SL, and the long-term necessity to delve into the impact of this methodology, the present investigation attempts to shed light in this sense and encourage scholars to contribute with further research on the field. However, due to some limitations, results should be approached with caution, since they are circumscribed to a specific context and sample. In addition, a pre-experimental study was used, although this is a typical research design in SL investigations (Yorio and Ye, 2012). In any case, further research should continue examining the longitudinal effects of SL (Maloney and Griffith, 2013; Chiva-Bartoll et al., 2019; Ruiz-Montero et al., 2019; Seban, 2019; Pérez-Ordás et al., 2021), and these studies could consider applying mixed methods in order to combine qualitative and quantitative perspectives.

Higher education is essential to guarantee sustainable development. In this sense, Seto-Pamies and Papaoikonomou (2020) think that universities can contribute to the development of SDGs from several perspectives:

-by providing students with the necessary abilities to act towards the achievement of SDGs (teaching function);

-by offering new formulas for the treatment of SDGs (research function); and -by communicating effectively and efficiently with the community, to collaborate and lead projects that will improve the environment (extension and university social responsibility function).

The present work may serve as an example of how to bring these ideals into practice, since it has shown how higher education can contribute to SDG development from these three perspectives. First, the civic attitudes and skills developed by students are essential to move forward towards SDG attainment. Second, research leads towards better understanding of methodological strategies promoting SDG, positing SL as an adequate possibility in this respect. Third, in order to carry out the programme, communication and coordination between all the agents involved (university professors, university students, relatives and social entities) are essential to plan the lines of action. The service provided to children with functional diversity has generated a positive impact on them and in the community. Thanks to this experience, these children were able to enjoy and develop their motor skills, while university students were able to gain experience which will be essential in their professional future in order to properly attend diversity in the school. This is not only interesting for their training because they will encounter these cases in their professional future, but also to serve as a platform for these students to know whether they want to devote part of their future training to specialisation in this area or, equally validly, whether they do not want to do so. Both decisions will be the result of a possibility of experience in their initial training, something that should be seen as a necessary implementation opportunity in the university environment. From this point of view, it is not only necessary to vindicate the social need that exists when discovering a need, but also to vindicate the existence of spaces in the university that serve as a platform to develop this type of project.

## Data Availability Statement

The datasets generated for this study are available on request to the corresponding author.

## Ethics Statement

The studies involving human participants were reviewed and approved by Universitat Jaume I Ethics committee. The patients/participants provided their written informed consent to participate in this study.

## Author Contributions

MM-V and JG-G: conceptualization, investigation, and supervision. MM-V, JG-G, and TV-E: methodology. TV-E and JG-G: validation and formal analysis. OC-B, TV-E, and CS-G: data curation. MM-V, JG-G, TV-E, OC-B, and CS-G: writing-original draft preparation. MM-V, CS-G, and OC-B: writing-review and editing. MM-V: project administration. OC-B and JG-G: funding acquisition. All authors have read and agreed to the published version of the manuscript.

## Funding

This research was funded by the Universitat Jaume I, research grant numbers UJI–A2019–01, PREDOC/2016/53, Conselleria de Innovación, Universidades, Ciencia y Sociedad Digital (Generalitat Valenciana, Best/2019/110), Fulbright Comission and University of Valencia grants.

## Conflict of Interest

The authors declare that the research was conducted in the absence of any commercial or financial relationships that could be construed as a potential conflict of interest.

## Publisher’s Note

All claims expressed in this article are solely those of the authors and do not necessarily represent those of their affiliated organizations, or those of the publisher, the editors and the reviewers. Any product that may be evaluated in this article, or claim that may be made by its manufacturer, is not guaranteed or endorsed by the publisher.

## Footnotes

^1^ https://www.iea.nl/studies/iea/iccs
